# Predicting Bevirimat resistance of HIV-1 from genotype

**DOI:** 10.1186/1471-2105-11-37

**Published:** 2010-01-20

**Authors:** Dominik Heider, Jens Verheyen, Daniel Hoffmann

**Affiliations:** 1 Department of Bioinformatics, Center of Medical Biotechnology, University of Duisburg-Essen, Universitaetsstr. 2, 45117 Essen, Germany; 2 Institute of Virology, University of Cologne, Fuerst-Pueckler-Str. 56, 50935 Cologne, Germany

## Abstract

**Background:**

Maturation inhibitors are a new class of antiretroviral drugs. Bevirimat (BVM) was the first substance in this class of inhibitors entering clinical trials. While the inhibitory function of BVM is well established, the molecular mechanisms of action and resistance are not well understood. It is known that mutations in the regions CS p24/p2 and p2 can cause phenotypic resistance to BVM. We have investigated a set of p24/p2 sequences of HIV-1 of known phenotypic resistance to BVM to test whether BVM resistance can be predicted from sequence, and to identify possible molecular mechanisms of BVM resistance in HIV-1.

**Results:**

We used artificial neural networks and random forests with different descriptors for the prediction of BVM resistance. Random forests with hydrophobicity as descriptor performed best and classified the sequences with an area under the Receiver Operating Characteristics (ROC) curve of 0.93 ± 0.001. For the collected data we find that p2 sequence positions 369 to 376 have the highest impact on resistance, with positions 370 and 372 being particularly important. These findings are in partial agreement with other recent studies. Apart from the complex machine learning models we derived a number of simple rules that predict BVM resistance from sequence with surprising accuracy. According to computational predictions based on the data set used, cleavage sites are usually not shifted by resistance mutations. However, we found that resistance mutations could shorten and weaken the *α*-helix in p2, which hints at a possible resistance mechanism.

**Conclusions:**

We found that BVM resistance of HIV-1 can be predicted well from the sequence of the p2 peptide, which may prove useful for personalized therapy if maturation inhibitors reach clinical practice. Results of secondary structure analysis are compatible with a possible route to BVM resistance in which mutations weaken a six-helix bundle discovered in recent experiments, and thus ease Gag cleavage by the retroviral protease.

## Background

### HIV and Bevirimat

Bevirimat (BVM) [1] belongs to a new class of anti-HIV substances that inhibit maturation of virus particles by preventing cleavage of precursor polyprotein by the retroviral protease (PR). BVM prevents the final cleavage of precursor protein p25 to p24 and p2, hence p25 proteins are accumulating in the immature virions. These immature viral particles are not capable of transforming to an infectious stage, and the viral replication cycle is interrupted. A first set of mutations conferring resistance to BVM were found in selection experiments with BVM and were located at CS p24/p2 [1-4]. In clinical phase II trials, polymorphisms in the QVT-motif of p2 were found to prevent antiretroviral activity of BVM and were extensively studied in phenotypic resistance assays [5-7].

### Machine learning

The notion of a *resistance mutation *is often useful as a first, simple approximation to describe relations between point mutations and resistance phenotypes. However, it is often observed that the more data become available the more complex are the relations between genotype and phenotype that show up. For instance, it has been observed that mutations in the QVT motif (wild type sequence 369-371) are preferentially associated with resistance to BVM [8]. However, as the data analyzed in the current study shows, the same set of mutations of QVT to QAS can be associated with BVM resistance [5] or susceptibility [6], depending on the mutational background. Machine learning methods are built to cope with such complex associations.

There are several machine learning methods that have been successfully employed to this end, e.g. rule-based methods [9], decision trees [10,11], support vector machines [12], random forests (RFs) [13], or artificial neural networks (ANNs) [14-16].

ANNs are universal approximators that can be used to solve non-linear classification problems; they are prone to overtraining if not properly set up [17,18]. RFs are also excellent non-linear models, and in general perform better than single decision trees (DTs) [19]. They are less easily interpretable than DTs, although they provide variable importance measures [20]. In contrast, rule based systems yield rules that are well intelligible, but often classify not optimally [21,22].

## Methods

### Data

Sequences of the p24/p2 region of 45 strains of HIV-1 with susceptibility or intermediate resistance to BVM (here defined as *IC*_50 _≤ 10) were used, and 110 sequences of resistant strains (*IC*_50 _*>*10). The phenotype was determined in experiments in which HIV-1 was cultured in the presence of increasing concentrations of BVM. The concentration of BVM inhibiting 50% of viral replication compared to cell culture experiments without BVM is defined as *IC*_50 _(50% inhibitor concentration). In general, drug resistance means reduced inhibition of viral replication by antiretroviral drugs, resulting in increased *IC*_50 _values. The *IC*_50 _values of the drug resistant isolates and HIV wild type are used to calculate resistance factors

a standardized measure of HIV drug resistance. The cut-off value of the resistance factor used to define the classes "resistant to BVM" and "susceptible to BVM" was previously derived from data obtained in phase II clinical trials with BVM correlating phenotypic resistance and clinical response [6,7].

All data were collected from several studies that have investigated polymorphisms in p2, especially in its C-terminal half [1,5-7] (see additional file 1 for complete set). Duplicated sequences in each class were removed prior to analysis.

#### Multiple Sequence Alignment

Multiple sequence alignments of the sequences were produced with clustalw [23], t-coffee [24], muscle [25], and prank [26]. Clustalw and muscle gave very compact alignments with a width of 21 columns and most rows free of gaps. The alignment from t-coffee was wider by one column, and the prank alignment much wider with 36 columns. Since clustalw and muscle gave similar alignments, and the prank alignment led to a relatively poor predictive performance, we restrict ourselves in the following to reporting results based on the output of clustalw and t-coffee (see additional files 2 and 3).

#### Descriptor set

It is often helpful to analyze not the sequences of amino acids as strings of characters, but to associate with each amino acid a numerical "descriptor" value, for instance a value that captures a physico-chemical property of this amino acid. Recently, it has been shown that the descriptor set is the most critical element in classification [27,28], and that physico-chemical descriptors outperform simpler descriptors [29]. In our search for a method with maximum predictive power we tested several numerical descriptors, including hydrophobicity values of Kyte and Doolittle [30], molecular weight, isoelectric point (IEP) and pKa values for each amino acid. Moreover, we used the predicted probability for cleavage by HIV protease as a descriptor [31]. The numerical descriptor values for gaps from the multiple sequence alignment are undefined *a priori*. We therefore tested three values for gaps, namely 0, -1 and an interpolated value (mean of the two amino acid descriptor values on both sides of gap). In the case of 0 and interpolated values for gaps the descriptor values of the amino acids were normalized to the interval [-1,1], and in the case of -1 for a gap they were normalized to [0,1]. Apart from using numerical descriptors, we also trained an RF with the multiply aligned p2 sequences using as factors the single letter codes of the amino acids and "-" for gaps.

#### Neural Networks

We used a Java implementation http://www.heatonresearch.com/encog of neural networks with one hidden layer and three to seven hidden neurons. Resilient propagation (Rprop) was applied as a learning rule [32]. We used the identity function as activation function for the input layer and the logistic function for the hidden and output layer, respectively. We have used the logistic function because it has been shown in recent studies that it leads to a better generalization ability [33,34]. The weights of the ANNs were initiated by applying the Nguyen-Widrow-method [35]. Stop-training was performed in order to avoid overfitting of the neural networks [36].

#### Random Forests

As an alternative to ANNs we trained Random Forests (RFs) [20] for the prediction of BVM resistance, using the implementation in the randomForest package of R [37]. In our application each RF consisted of 2000 randomly and independently grown decision trees. When using the trained RF for prediction, an unseen sequence was assigned to the resistance class voted for by at least 50% of the trees. The importance of each variable, i.e. sequence position, for the correct classification can be assessed by determining the increase in misclassification rate due to leaving this variable [20]. Furthermore, we used the rpart package of R [37] to create single decision trees.

#### Rule-based systems

We used the rule-based algorithms JRip [38] and PART [39] as implemented in R [37].

#### Cross-validation

All machine learning methods were validated using 100-fold leave-one-out [40] validation to assess for the different machine learning methods the mean prediction sensitivity, specificity, and accuracy (see formulas below) and the ability to generalize to unseen sequences. In addition to this cross-validation, we report for RFs an out-of-bag (OOB) error, an upper limit of the classification error [20].

**Figure 1:**
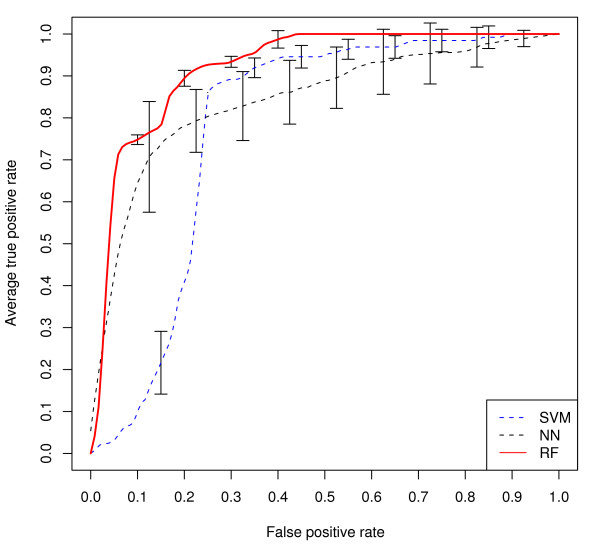
**ROC curve**. Averaged ROC curves of the best performing descriptor for each (non-discrete) machine learning approach. The standard deviation bars mark the 95% confidence intervals. blue: SVM; green: ANN; red: RF.

For each test in the cross-validation, the sensitivity, specificity, and accuracy were calculated according to:

with true positives *TP*, false positives *FP*, false negatives *FN *and true negatives *TN*. Figure 1 shows sensitivities and specificities as ROC curves (Receiver Operating Characteristics) [41] for the non-discrete methods in our study. Table 1 gives the corresponding areas under the curve (AUC). ROC curves were drawn with R-package ROCR [42].

**Table 1:** Area under the curve.

method	descriptor	mean AUC	sd	cv
RF	hydrophobicity	0.927	0.001	0.001
	molecular weight	0.923	0.001	0.001
	IEP	0.909	0.001	0.001
	pKa	0.914	0.001	0.001
	cleavage site prediction	0.851	0.003	0.003

ANN	hydrophobicity	0.841	0.028	0.034
	molecular weight	0.839	0.022	0.026
	IEP	0.721	0.036	0.050
	pKa	0.733	0.028	0.038
	cleavage site prediction	0.762	0.036	0.047

linear model	hydrophobicity	0.826	0.008	0.009
	molecular weight	0.811	0.000	0.000
	IEP	0.784	0.000	0.000
	pKa	0.777	0.000	0.000
	cleavage site prediction	0.803	0.000	0.000

decision tree	hydrophobicity	0.815	0.000	0.000
	molecular weight	0.841	0.000	0.000
	IEP	0.771	0.000	0.000
	pKa	0.764	0.000	0.000
	cleavage site prediction	0.803	0.000	0.000

JRip	hydrophobicity	0.825	0.000	0.000
PART	hydrophobicity	0.890	0.000	0.000
Rule372	hydrophobicity	0.710	0.000	0.000

Results of the 100-fold leave-one-out validation. The *pro forma *AUC values for the discrete methods (decision trees and rule based models) are just for comparison purposes. sd: standard deviation; cv: coefficient of variation.

### Structure and cleavage-site prediction

Secondary structures of all p2 sequences of 20 or more residues were predicted using JPred [43]. Based on statistical evidence, the secondary structure predictions did also yield a reliability index from 0 (unreliable) through 9 (highly reliable) for each residue being in a predicted secondary structure state.

HIV protease cleavage sites for all p2 sequences were predicted with HIVcleave [31] based on earlier work by Chou *et al*. [44].

### Statistical comparison

All models were compared by applying Wilcoxon Signed-Rank test [45] on the AUC distributions from the 100-fold leave-one-out cross-validation runs [46]. The null hypothesis was that there are no differences between the compared classifiers.

## Results and Discussion

### Prediction performance of machine learning methods

All machine learning methods were trained in various configurations and with several descriptors as described in methods. The prediction qualities, such as the mean AUCs (), standard deviation (sd) and coefficient of variation (*cv *= ) are shown in Table 1.

The ANNs yielded AUCs between 0.72 ± 0.036 (descriptor IEP) and 0.84 ± 0.028 (descriptor hydrophobicity). According to the Wilcoxon Signed-Rank test [46] with significance level *α *= 0.001 the mean AUC for descriptor molecular weight was not significantly different from that obtained with descriptor hydrophobicity, while all other descriptors gave significantly lower values of mean AUC. There were no significant differences (*α *= 0.001) between the mean AUCs of each descriptor with regard to the number of hidden neurons.

RFs performed consistently better than ANNs for all descriptors, reaching AUC values between 0.85 ± 0.003 (cleavage site prediction) and 0.93 ± 0.001 (hydrophobicity). Again, the best results, with only small differences, were obtained from hydrophobicity and molecular weight as descriptors. The OOB error with this descriptor was 7.59%. For comparison, the best single decision tree, which was created with rpart in R [37], reached a *pro forma *AUC of 0.841 (see Table 1).

**Figure 2:**
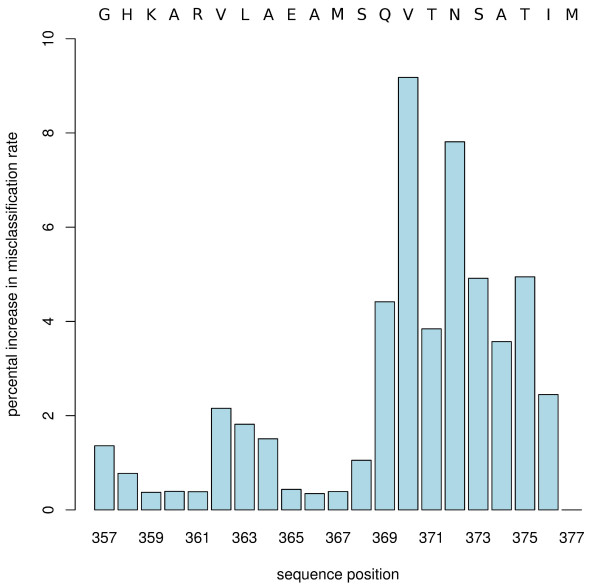
**Importance of sequence positions in RF predictions**. Importance of sequence positions in p2 for prediction of BVM resistance by RFs. The y-axis denotes the "percental increase in misclassification rate" [20]. The upper horizontal axis indicates wild type sequence.

The RFs find the most important sequence positions for resistance prediction in the second half of the p2 sequence, especially at sequence positions 369-376 (Figure 2) in the clustalw alignment; in the wild type sequence this region corresponds to the motif QVTNSATI. The two positions 370 (V in wild type) and 372 (N in wild type) have by far the highest importance in the investigated data set. This finding is in partial agreement with the findings of other workers who identified the QVT motif at positions 369-371 as important [7]. Positions 363 and 364 are not as prominent in terms of importance, although they were previously identified as crucial [47] for resistance to BVM. The apparently lower importance of these positions in the current study can be explained by the nature of our data set, which focuses on resistance mediated by baseline mutations within the p2 region in clinical HIV isolates.

We also trained RFs on the actual sequences, i.e. without numerical descriptors. These RFs gave OOB errors above those trained with hydrophobicities, namely of 13.55% for the t-coffee alignment and 14.84% for the clustalw alignment. For comparison, other machine learning methods were tested as well, including Hidden Markov Models (HMMs) [48] and linear models. We tested linear support vector machines (SVMs) and logistic regression as implemented in R [37], and furthermore, simple perceptrons implemented in Java http://www.heatonresearch.com/encog. All of these models performed worse compared to the RFs. The best linear model (AUC 0.826 ± 0.008) was a linear SVM using hydrophobicity as a descriptor. In Table 1 we report the results of the best linear model for each descriptor. The HMMs were not able to classify the sequences. In Figure 1 the ROC-curves for the descriptors performing best for each (non-discrete) machine learning method are shown.

### Genotype-phenotype rules

The two algorithms JRip and PART for rule extraction provided rule sets that performed well in the cross-validation with accuracies reaching almost that of RFs. Since the rules derived from the t-coffee alignment had lower errors than those based on the clustalw alignment, we here report only the former rules.

During cross-validation JRip generated most frequently a set of three rules relating alignment positions, hydrophobicities, and BVM resistance class. Translated to amino acid residues, the rules are:

1. IF position 370 ∈ {*I*, *V*} AND NOT position 373 ∈ {*K*, *R*} AND position 374 ∈ {*I*, *V*, *L*, *F*, *C*, *M*, *A*} ⇒ susceptible

2. IF position 372 ∈ {*K*, *R*} AND position 373 ∈ {*P*, *H*, *E*, *N*, *Q*, *D*} ⇒ susceptible

3. ELSE resistant

JRip reaches in the cross-validation a mean sensitivity of 77.01% at a specificity of 88.14%. Dropping the first rule leads to a sensitivity of 11.76% and a specificity of 99.21%. Dropping the second rule leads to a sensitivity of 72.54% with a corresponding specificity of 88.1%.

In the cross-validation PART most frequently extracted fifteen rules (see additional file 4) with a sensitivity of 85.5% and a specificity of 93.27%. Remarkably, the PART rules did take exactly those sequence positions into account that had non-zero importance in the RF analysis (see Figure 2). As suggested by the JRip and PART rules, resistance is generally caused by patterns of two or more residues. However, the importance plot (Figure 2) show that single positions may be useful indicators as well. E.g. we found that at sequence position 372 the hydrophobicity values of resistant and susceptible group clustered around two different values, 0.39 for the resistant and 0.26 for the susceptible. From this we could derive the rule (Rule372): a sequence is resistant if the hydrophobicity at 372 is closer to the mean hydrophobicity of the resistant cluster than to that of the susceptible cluster and vice versa. The rule is predictive with 52% sensitivity and 90% specificity.

### Structural and functional implications of resistance mutations

After experiments [49] have excluded the classical molecular mechanism of protease inhibition, i.e. blocking of its catalytic site, there are still several molecular mechanisms for BVM action considered in the literature (for review see [1]): BVM could directly occlude the protease cleavage site ("direct" mechanisms, possibly with contact of BVM and protease), or it could stabilize a Gag structure that has to be weakened or dissolved to make the cleavage sites accessible to the protease ("indirect" mechanisms, possibly *without *contact of BVM and protease). Accordingly, there are several possible resistance mechanisms discussed in the literature, such as mutations that perturb the BVM binding site, that weaken the mentioned Gag structure, or that make the affected cleavage site easier digestible for the protease. A hypothetical resistance mechanism that to our knowledge so far has not been addressed is a shift of the cleavage site. We have therefore investigated associations of resistance mutations with cleavage site locations properties, as predicted computationally. In all susceptible and most resistant sequences the predicted PR cleavage sites with maximum probability were unchanged with respect to the wild type (see additional file 5): cleavage was predicted to be most probable at P_1_-sites 363 and 367 in agreement with experimental findings [50], and cleavage probabilities at P_1 _363 were rather invariable across the data set. In a few resistant sequences cleavage sites probabilities were indeed predicted to shift (see additional file 6). Amongst these sequences we observed a tendency for the second cleavage site at P_1 _367 to have lower probabilities whereas position 365 did emerge as a new possible P_1 _site. However, since this occurs rather rarely, the data do not support a shift of the cleavage site as a major resistance mechanism. It is notable that in the studied data the positions 372-376 most relevant for resistance (Figure 2) lie outside the protease binding region P_4_-P_4_' for P1 at 363 (P_4_' 367). Even for the internal cleavage site at P_1 _367 (P_4_' 371), more than half of these important positions are outside the protease binding site. This finding is consistent with a model that allows for an "indirect" mechanism of BVM, though it cannot exclude "direct" mechanisms. In fact, mutations found in other studies closer to the cleavage sites [47,49] also allow for a direct model.

**Figure 3:**
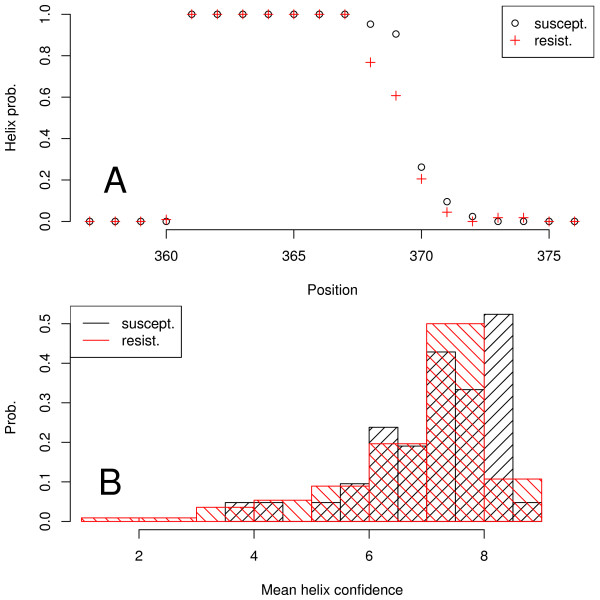
**Helix length and confidence**. Secondary structure predictions for p2 with susceptibility and resistance to BVM. A: For all p2 sequences the secondary structure was predicted by JPred [43] and then for each sequence position the helix probability (fraction of helix at this position) was computed separately for the susceptible and resistant sequences. B: Histograms of the confidence with which JPred predicts a helix (0 lowest, 9 highest). The confidence values were averaged for each sequence over all positions predicted to be helical.

A key component of an indirect mechanism is a structure within Gag that has to be weakened prior to cleavage of p24/p2. A candidate structure is the *α*-helix first predicted by Accola *et al*. [50]. We have extended secondary structure predictions to all sequences of the data set, including the wild type. All these structures were predicted as mainly *α*-helical in the central part (additional file 7). This gross feature is consistent with the experimental structure by Morellet *et al*. [51], though the predicted helices are shorter. While in the Morellet structure the helix comprises all of the residues starting at position 358, the predicted helices comprise between seven and twelve of the 21 sequence positions and typically start at position 361 (Figure 3A). Apart from the deficiencies of the prediction method the difference between experiment and prediction may be due in part to the experimental conditions [51] where a substantial amount of trifluoroethanol in the solution could have led to a helix content exceeding that in the native state. The earlier work by Worthylake *et al*. [52] supports the view that the helix formed by p2 as such is not very stable. A very stable helix at the cleavage site could possibly prevent PR from cleaving, because the protease requires its substrate in an extended conformation [53]. On the other hand, recent data from electron microscopy [54] are compatible with bundles of six p2 helices stabilizing the immature matrix of the virus. In summary predictions and experiments point to a weak p2 helix that is stabilized by its environment.

It is remarkable that the end of the predicted helices around position 369 coincides with start of the sequence region most important for resistance (Figure 2) in our data set; in other words, the sequence positions most important for resistance in our data lie outside the predicted *α*-helix in a region of unspecified secondary structure. Moreover, the resistant sequences have a tendency for shorter helices compared to susceptible sequences, as can be seen in the earlier drop of helix probability at around position 368 in Figure 3A.

We have also analyzed the confidence with which the secondary structure prediction algorithm assigns residues to a helical state. If we assume that the prediction is based on a representative sample of sequences observed as helices and non-helices, respectively, then this confidence could have a positive correlation with helix stability. A comparison of resistant and susceptible sequences with respect to mean confidence along the helix shows that resistant sequences have a tendency to lower helix confidence, and, if the assumption holds, lower helix stability (Figure 3B).

The above tendencies of resistance class, predicted helix length and confidence may reflect possible "indirect" resistance mechanisms: shorter and weaker helices could limit the effect of BVM in several ways, e.g. by destabilizing the binding site of BVM that may lie on the six-helix bundle mentioned above, or by easing the unraveling of the remaining helix, and thus cleavage by PR in presence of BVM. This argument suggests to test whether helix length and helix confidence are predictive of resistance. We have therefore trained another random forest solely with the predicted helix lengths and confidences, and without reference to the detailed sequences. This random forest had an OOB error of 23%, which is not as good as the errors of 8-15% reported above for random forests or rule-based methods trained on sequence information, but still much better than random guessing. This means that tuning of helicality of p2 could indeed be a BVM resistance mechanism.

## Conclusions

BVM was the first drug of the new class of maturation-inhibitors of HIV-1 that has reached phase II clinical trials. Several polymorphisms in p2 of HIV-1 hampered the sustained suppression of viral replication in these patients and conferred phenotypic resistance [7]. Since these polymorphisms were found in about 30% of treatment naïve HIV isolates and were significantly accumulated in PI resistant HIV isolates [55], genotypic resistance testing seems to be mandatory before administration of BVM.

Our analysis has shown that with the available sequences and corresponding phenotypic data it is possible to train machine learning methods that predict phenotypic resistance to BVM, mediated by baseline mutations of the p2 region, for unseen sequences with an error of less than 10%. This result is compatible with the view that mutations in p2 are the main reason for BVM resistance observed in clinical isolates not responding to BVM in clinical phase I and II studies. The high classification accuracy is encouraging for personalized therapy based on genotypical testing in case BVM-like drugs will become part of the antiretroviral repertoire. With a larger, representative data set of genotype-phenotype pairs, it could become feasible to use machine learning methods not only for classification but also for regression, i.e. prediction of quantitative resistance factors.

Random forests, the method with the best classification results amongst those tested, also allowed for the identification of the sequence positions most relevant for resistance. In our data set, these sequence positions cluster in the C-terminal half of p_2_, mostly outside the P_4_-P_4_' protease binding region. This is in agreement with the outcome of rule-based methods.

As judged from predicted cleavage positions, resistance mutations do usually not shift the cleavage site. Secondary structure prediction shows that resistance mutations may affect the length and strength of the *α*-helix formed by at least sequence positions 371-377 and covering also the cleavage site. This hypothesis is in agreement with propositions by other workers [1] and suggests possible resistance mechanisms that also may occur in combination, e.g. (a) resistance mutations could destroy the BVM binding site that may lie in the C-terminal half of p2, formed by several p2 peptides in the six-helix bundle suggested by Wright *et al*. [54]; (b) resistance mutations could weaken the *α*-helix in p2, and thus, the six-helix bundle in the immature virus. This could ease unraveling of the helix prior to cleavage by PR, and hence, may functionally outweigh a stabilizing effect of BVM on the helix bundle.

## Authors' contributions

All authors have jointly developed the research concept and collaborated on the writing of the manuscript. DH* has carried out computational analyses and drafted the manuscript. JV has initiated the study and revised the manuscript. DH has interpreted results and revised the manuscript.

All authors read and approved the final manuscript.

## Supplementary Material

Additional file 1 **Data set**. The sequences used in this study. Click here for file

Additional file 2 **MSA of the sequences with clustalw**. Multiple sequence alignment of the sequences with clustalw [23]. Click here for file

Additional file 3 **MSA of the sequences with t-coffee**. Multiple sequence alignment of the sequences with t-coffee [24]. Click here for file

Additional file 4 **Plots and rules**. Variance plots and prediction rules. Click here for file

Additional file 5 **Cleavage site predictions**. Predictions are made with HIVcleave [31]. Click here for file

Additional file 6 **Shifted cleavage site probabilities**. Probable HIV-protease cleavage sites are shown in bold [31]. The value represents the probability of protease cleavage. Click here for file

Additional file 7 **Secondary structure predictions**. Predictions are made with JPred [43]. Click here for file

## References

[B1] Salzwedel K Martin D Sakalian M Maturation inhibitors: a new therapeutic class targets the virus structure AIDS Rev 2007 9 162 172 17982941

[B2] Adamson CS Ablan SD Boeras I Goila-Gaur R Soheilian F Nagashima K Li F Salzwedel K Sakalian M Wild CT Freed EO In vitro resistance to the human immunodeficiency virus type 1 maturation inhibitor PA-457 (Bevirimat) J Virol 2006 80 22 10957 10971 10.1128/JVI.01369-06 16956950 PMC1642185

[B3] Li F Zoumplis D Matallana C Kilgore N Reddick M Yunus A Adamson C Salzwedel K Martin D Allaway G Freed E Wild C Determinants of activity of the HIV-1 maturation inhibitor PA-457 Virology 2006 356 217 24 10.1016/j.virol.2006.07.023 16930665

[B4] Adamson CS Waki K Ablan SD Salzwedel K Freed EO Impact of human immunodeficiency virus type 1 resistance to protease inhibitors on evolution of resistance to the maturation inhibitor bevirimat (PA-457) J Virol 2009 83 10 4884 4894 10.1128/JVI.02659-08 19279107 PMC2682084

[B5] Margot N Gibbs C Miller M Phenotypic susceptibility to Bevirimat among HIV-infected patient isolates without prior exposure to Bevirimat Proceedings of the 16th Conference on Retroviruses and Opportunistic Infections, Montreal, Canada 2009

[B6] Salzwedel K Harmy F Louvel S Sakalian M Reddick M Finnegan C Martin D McCallister S Klimkait T Allaway G Susceptibility of diverse HIV-1 patient isolates to the maturation inhibitor, Bevirimat (MPC-4326), is determined by clade-specific polymorphisms in Gag CA-SP1 Proceedings of the 16th Conference on Retroviruses and Opportunistic Infections, Montreal, Canada 2009

[B7] Baelen KV Salzwedel K Rondelez E Eygen VV Vos SD Verheyen A Steegen K Verlinden Y Allaway GP Stuyver LJ Susceptibility of human immunodeficiency virus type 1 to the maturation inhibitor bevirimat is modulated by baseline polymorphisms in Gag spacer peptide 1 Antimicrob Agents Chemother 2009 53 2185 2188 10.1128/AAC.01650-08 19223634 PMC2681549

[B8] McCallister S Lalezari J Richmond G Thompson M Harrigan R Martin D Salzwedel K Allaway G HIV-1 Gag polymorphisms determine treatment response to bevirimat (PA-457) Antivir Ther 2008 13 Suppl 3 A10

[B9] Lathrop R Steffen N Raphael M Deeds-Rubin S Pazzani M Cimoch P See D Tilles J Knowledge-based avoidance of drug-resistant HIV mutants AI MAGAZINE 1999 20 1 13 25

[B10] Sevin AD DeGruttola V Nijhuis M Schapiro JM Foulkes AS Para MF Boucher CAB Methods for Investigation of the Relationship between Drug-Susceptibility Phenotype and Human Immunodeficiency Virus Type 1 Genotype with Applications to AIDS Clinical Trials Group 333 J Infect Dis 2000 182 59 67 10.1086/315673 10882582

[B11] Beerenwinkel N Schmidt B Walter H Kaiser R Lengauer T Hoffmann D Korn K Selbig J Diversity and complexity of HIV-1 drug resistance: a bioinformatics approach to predicting phenotype from genotype Proc Natl Acad Sci USA 2002 99 12 8271 8276 10.1073/pnas.112177799 12060770 PMC123057

[B12] Beerenwinkel N Schmidt B Walter H Kaiser R Lengauer T Hoffmann D Korn K Selbig J Geno2pheno: Interpreting Genotypic HIV Drug Resistance Tests IEEE Intelligent Systems 2001 16 35 41 10.1109/5254.972080

[B13] Murray RJ Lewis FI Miller MD Brown AJ Genetic basis of variation in tenofovir drug susceptibility in HIV-1 AIDS 2008 22 10 1113 23 10.1097/QAD.0b013e32830184a1 18525256

[B14] Resch W Hoffman N Swanstrom R Improved success of phenotype prediction of the human immunodeficiency virus type 1 from envelope variable loop 3 sequence using neural networks Virology 2001 288 51 62 10.1006/viro.2001.1087 11543657

[B15] Draghici S Potter RB Predicting HIV drug resistance with neural networks Bioinformatics 2003 19 98 107 10.1093/bioinformatics/19.1.98 12499299

[B16] Wang D Larder B Enhanced prediction of lopinavir resistance from genotype by use of artificial neural networks J Infect Dis 2003 188 5 653 660 10.1086/377453 12934180

[B17] King R Feng C Sutherland A Comparison of classification algorithms on large real-world problems Applied Artificial Intelligence 1995 9 3 259 287 10.1080/08839519508945477

[B18] Tzafestas S Dalianis PJ Anthopoulos G On the overtraining phenomenon of backpropagation neural networks Mathematics and computers in simulation 1996 40 505 663 10.1016/0378-4754(96)90015-4

[B19] Banfield RE Hall LO Bowyer KW Kegelmeyer WP A comparison of decision tree ensemble creation techniques IEEE Transactions on Pattern Analysis and Machine Intelligence 2007 29 1 173 180 10.1109/TPAMI.2007.250609 17108393

[B20] Breiman L Random Forests Machine Learning 2001 45 5 32 10.1023/A:1010933404324

[B21] Kingston J Rule-based expert systems and beyond: an overview British Association of Accountants' Conference 1987

[B22] Witten IH Frank E Data Mining. Morgan Kauffmann 2000

[B23] Thompson J Higgins D Gibson T CLUSTAL W: improving the sensitivity of progressive multiple sequence alignment through sequence weighting, position-specific gap penalties and weight matrix choice Nucleic Acids Res 1994 22 4673 4680 10.1093/nar/22.22.4673 7984417 PMC308517

[B24] Notredame C Higgins DG Heringa J T-Coffee: A novel method for fast and accurate multiple sequence alignment J Mol Biol 2000 302 205 217 10.1006/jmbi.2000.4042 10964570

[B25] Edgar RC MUSCLE: a multiple sequence alignment method with reduced time and space complexity BMC Bioinformatics 2004 5 113 10.1186/1471-2105-5-113 15318951 PMC517706

[B26] Löytynoja A Goldman N An algorithm for progressive multiple alignment of sequences with insertions Proc Natl Acad Sci USA 2005 102 30 10557 10562 10.1073/pnas.0409137102 16000407 PMC1180752

[B27] Ong S Lin H Chen Y Li Z Cao Z Efficacy of different protein descriptors in predicting protein functional families BMC Bioinformatics 2007 8 300 10.1186/1471-2105-8-300 17705863 PMC1997217

[B28] Kernytsky A Rost B Using genetic algorithms to select most predictive protein features Proteins 2009 75 75 88 10.1002/prot.22211 18798568

[B29] Nanni L Lumini A Using ensembles of classifiers for predicting HIV protease cleavage sites in proteins Amino Acids 2009 36 409 416 10.1007/s00726-008-0076-z 18401541

[B30] Kyte J Doolittle R A simple method for displaying the hydropathic character of a protein J Mol Biol 1982 157 105 132 10.1016/0022-2836(82)90515-0 7108955

[B31] Shen HB Chou KC HIVcleave: a web-server for predicting human immunodeficiency virus protease cleavage sites in proteins Analytical Biochemistry 2008 375 388 390 10.1016/j.ab.2008.01.012 18249180

[B32] Riedmiller M Braun H A direct adaptive method for faster backpropagation learning: The Rprop algorithm Proceedings of the IEEE International Conference on Neural Networks 1993 586 591 full_text

[B33] Borschbach M Hauke S Pyka M Heider D Opportunities and limitations of a principal component analysis optimized machine learning approach for the identification and classification of cancer involved proteins Communications of the SIWN 2009 6 85 89

[B34] Heider D Appelmann J Bayro T Dreckmann W Held A Winkler J Barnekow A Borschbach M A computational approach for the identification of small GTPases based on preprocessed amino acid sequences Technology in Cancer Research and Treatment 2009 8 5 333 342 19754209 10.1177/153303460900800503

[B35] Nguyen D Widrow B Improving the learning speed of 2-layer neural networks by choosing initial values of the adaptive weights Proceedings of Intl Joint Conf on Neural Networks 1990 21 26 full_text

[B36] Punta M Rost B Neural networks predict protein structure and function Humana Press, Berlin, Germany 2008 chap. Artificial Neural Networks: Methods and Protocols 10.1007/978-1-60327-101-1_11 19065812

[B37] R Development Core Team R: A Language and Environment for Statistical Computing 2006 R Foundation for Statistical Computing, Vienna, Austria http://www.R-project.org ISBN 3-900051-07-0

[B38] Cohen WW Prieditis A, Russell S Fast effective rule induction Proceedings of the 12th International Conference on Machine Learning 1995 115 123

[B39] Frank E Witten IH Shavlik J Generating accurate rule sets without global optimization Machine Learning: Proceedings of the Fifteenth International Conference 1998

[B40] Cawley GC Leave-One-Out Cross-Validation Based Model Selection Criteria for Weighted LS-SVMs Proceedings of the IEEE World Congress on Computational Intelligence 2006

[B41] Fawcett T An introduction to ROC analysis Pattern Recognition Letters 2006 27 861 874 10.1016/j.patrec.2005.10.010

[B42] Sing T Sander O Beerenwinkel N Lengauer T ROCR: visualizing classifier performance in R Bioinformatics 2005 21 20 3940 3941 10.1093/bioinformatics/bti623 16096348

[B43] Cole C Barber JD Barton GJ The Jpred 3 secondary structure prediction server Nucleic Acids Res 2008 36 W197 201 10.1093/nar/gkn238 18463136 PMC2447793

[B44] Chou KC Tomasselli AG Reardon IM Heinrikson RL Predicting human immunodeficiency virus protease cleavage sites in proteins by a discriminant function method Proteins 1996 24 51 72 10.1002/(SICI)1097-0134(199601)24:1<51::AID-PROT4>3.0.CO;2-R 8628733

[B45] Wilcoxon F Individual comparisons by ranking methods Biometrics 1945 1 80 83 10.2307/3001968

[B46] Demsar J Statistical comparisons of classifiers over multiple data sets Journal of Machine Learning Research 2006 7 1 30

[B47] Zhou J Chen CH Aiken C Human immunodeficiency virus type 1 resistance to the small molecule maturation inhibitor 3-O-(3',3'-dimethylsuccinyl)-betulinic acid is conferred by a variety of single amino acid substitutions at the CA-SP1 cleavage site in Gag J Virol 2006 80 24 12095 101 10.1128/JVI.01626-06 17035324 PMC1676313

[B48] Eddy SR Profile hidden Markov models Bioinformatics 1998 14 9 755 63 10.1093/bioinformatics/14.9.755 9918945

[B49] Li F Goila-Gaur R Salzwedel K Kilgore NR Reddick M Matallana C Castillo A Zoumplis D Martin DE Orenstein JM Allaway GP Freed EO Wild CT PA-457: a potent HIV inhibitor that disrupts core condensation by targeting a late step in Gag processing Proc Natl Acad Sci USA 2003 100 23 13555 60 10.1073/pnas.2234683100 14573704 PMC263852

[B50] Accola MA Höglund S Göttlinger HG A putative alpha-helical structure which overlaps the capsid-p2 boundary in the human immunodeficiency virus type 1 Gag precursor is crucial for viral particle assembly J Virol 1998 72 2072 2078 9499062 10.1128/jvi.72.3.2072-2078.1998 PMC109501

[B51] Morellet N Druillennec S Lenoir C Bouaziz S Roques B Helical structure determined by NMR of the HIV-1 (345-392)Gag sequence, surrounding p2: Implications for particle assembly and RNA packaging Protein Science 2004 14 375 386 10.1110/ps.041087605 PMC2253411 15659370

[B52] Worthylake DK Wang H Yoo S Sundquist WI Hill CP Structures of the HIV-1 capsid protein dimerization domain at 2.6 A resolution Acta Crystallogr D Biol Crystallogr 1999 55 85 92 10.1107/S0907444998007689 10089398

[B53] Miller M Schneider J Sathyanarayana BK Toth MV Marshall GR Clawson L Selk L Kent SB Wlodawer A Structure of complex of synthetic HIV-1 protease with a substrate-based inhibitor at 2.3 A resolution Science 1989 246 4934 1149 52 10.1126/science.2686029 2686029

[B54] Wright ER Schooler JB Ding HJ Kieffer C Fillmore C Sundquist WI Jensen GJ Electron cryotomography of immature HIV-1 virions reveals the structure of the CA and SP1 Gag shells EMBO J 2007 26 8 2218 26 10.1038/sj.emboj.7601664 17396149 PMC1852790

[B55] Verheyen J Verhofstede C Knops E Vandekerckhove L Fun A Brunen D Dauwe K Wensing A Pfister H Kaiser R Nijhuis M High prevalence of bevirimat resistance mutations in protease inhibitor-resistant HIV isolates AIDS 2009 in press 19926962 10.1097/QAD.0b013e32833160fa

