# Supplementary material for: Predicting Bevirimat resistance of HIV-1 from genotype
Source: BMC Bioinformatics. 2010 Jan 20;11:37. doi: 10.1186/1471-2105-11-37 (PMC3224585; doi:10.1186/1471-2105-11-37)
Supplement: Additional file 4 — Plots and rules. Variance plots and prediction rules. [file 1471-2105-11-37-S4.DOC]

**PART**

PART decision list

------------------

369 <= 0.111111 AND

370 <= 0.7 AND

376 > -1 AND

357 > 0.411111: 0 (78.0)

363 > 0.811111 AND

364 <= 0.7 AND

368 <= 0.411111 AND

369 > 0.111111: 1 (6.0)

363 > 0.811111 AND

364 > 0.7: 0 (5.0)

363 <= 0.811111: 0 (4.0)

368 > 0.411111: 0 (4.0/1.0)

362 > 0.966667 AND

375 <= 0.422222: 1 (3.0/1.0)

362 <= 0.966667 AND

373 <= 0.322222 AND

369 > 0.066667 AND

371 <= -1 AND

372 <= 0.144444 AND

377 > -1 AND

376 > 0.966667: 1 (7.0)

362 <= 0.966667 AND

357 > 0.411111 AND

369 > 0.066667 AND

V19 <= 0.455556 AND

371 <= -1: 1 (11.0)

377 > -1 AND

362 <= 0.966667 AND

369 > 0.066667 AND

371 <= -1 AND

376 > -1 AND

372 <= 0.144444: 0 (8.0)

362 <= 0.966667 AND

369 > 0.066667 AND

373 <= 0.322222: 1 (9.0)

362 <= 0.966667 AND

372 <= 0.411111 AND

357 > 0.411111 AND

370 > 0.711111 AND

374 > 0.455556: 1 (7.0/2.0)

357 > 0.411111: 0 (5.0)

362 <= 0.966667 AND

372 > 0.411111: 0 (3.0)

362 > 0.966667: 0 (3.0)

: 1 (2.0)

Number of rules = 15


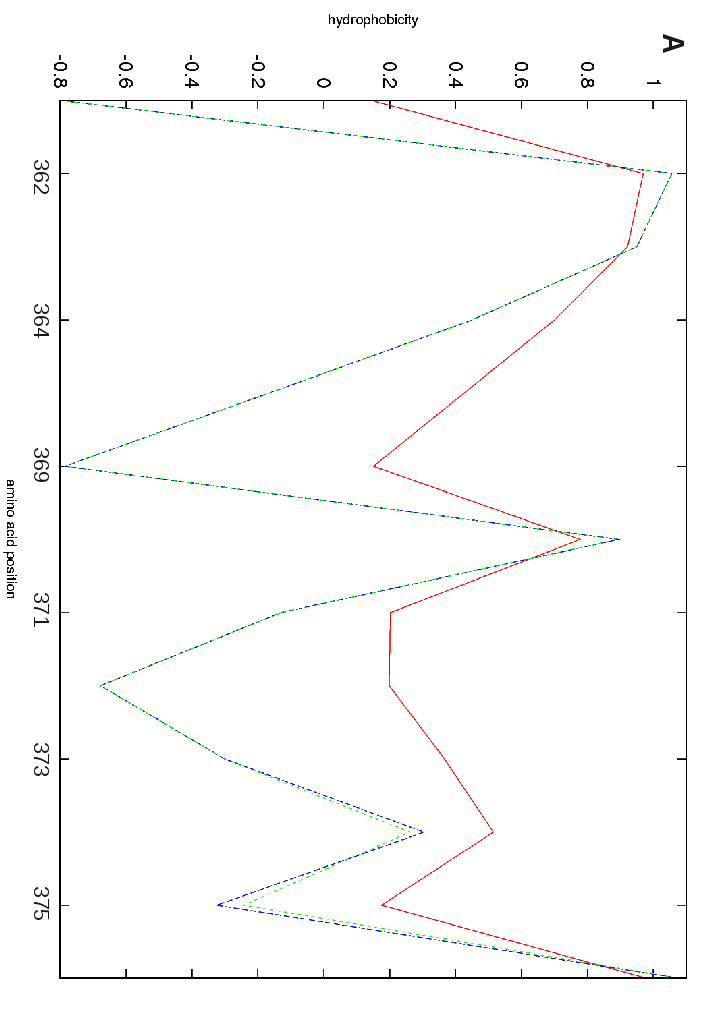


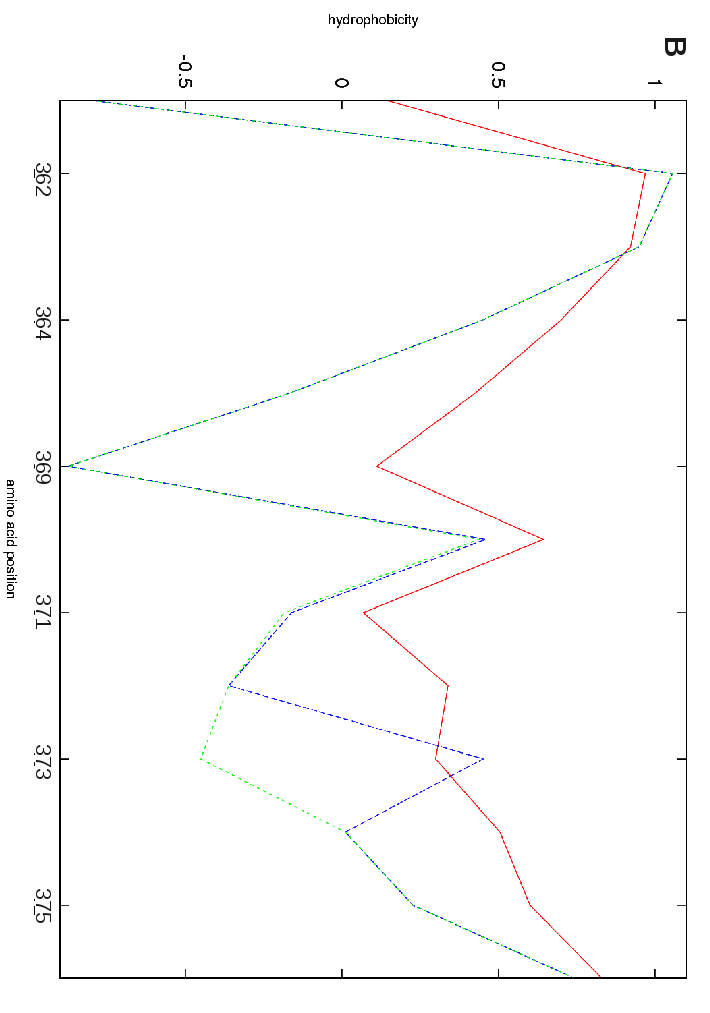


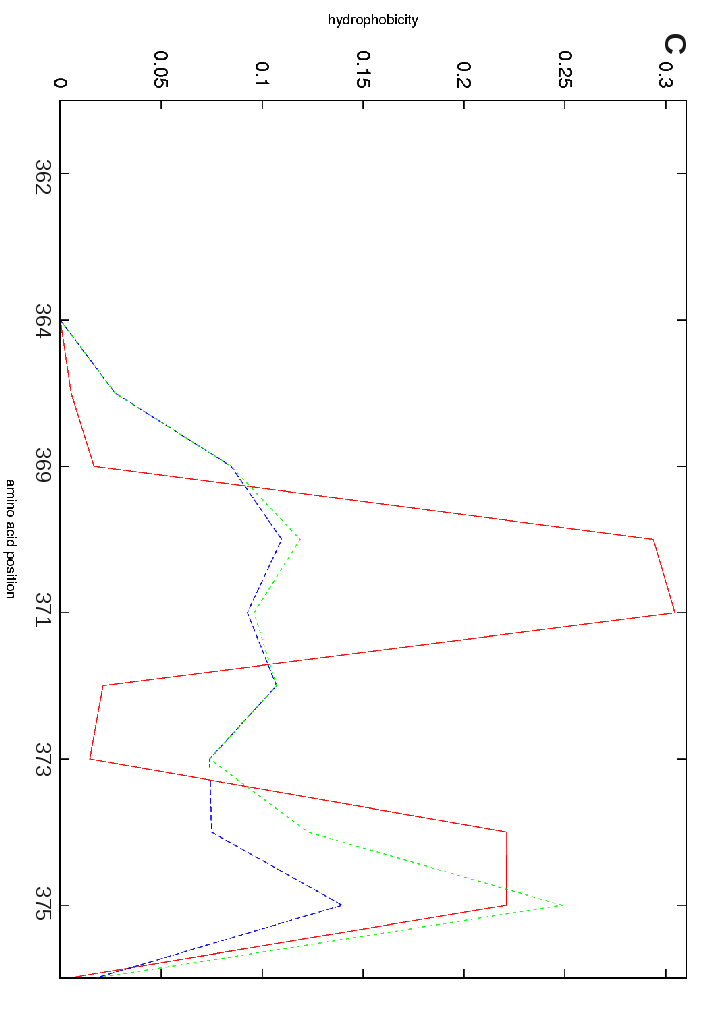


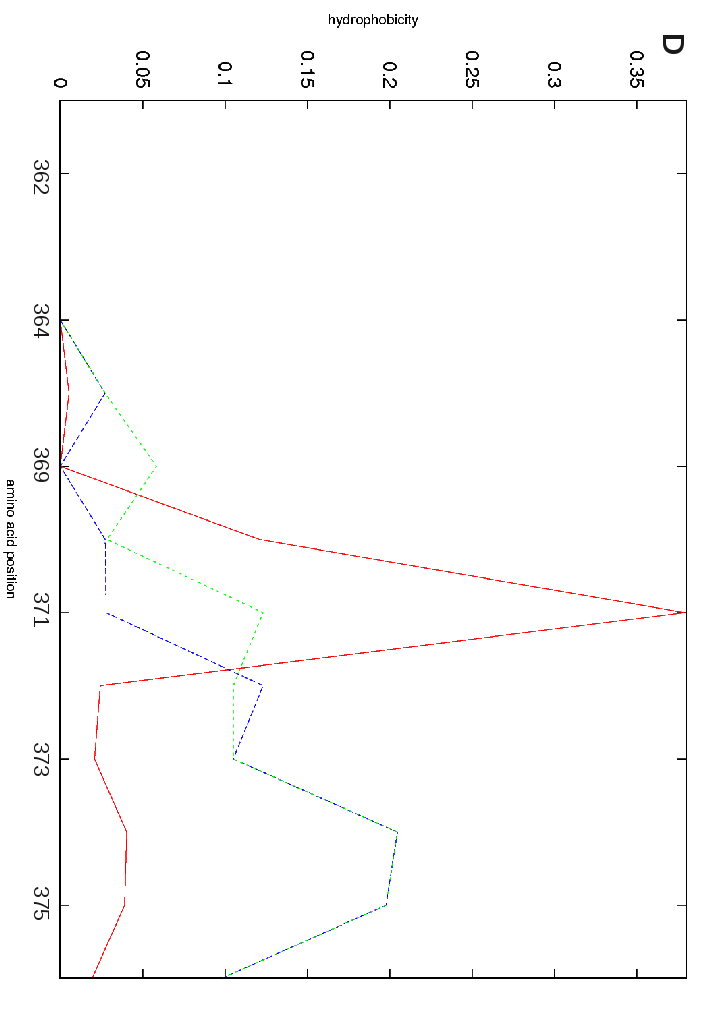


The mean and the variances are calculated for each position within the sequence.

red: deletion value=-1; blue: deletion value=0; green: interpolated deletion value;
A: mean of the susceptible sequences; B: mean of the resistant sequences; C: variance of the susceptible sequences; D: variance of the resistant sequences.
